# Evolution and phylogeny of the mud shrimps (Crustacea: Decapoda) revealed from complete mitochondrial genomes

**DOI:** 10.1186/1471-2164-13-631

**Published:** 2012-11-16

**Authors:** Feng-Jiau Lin, Yuan Liu, Zhongli Sha, Ling Ming Tsang, Ka Hou Chu, Tin-Yam Chan, Ruiyu Liu, Zhaoxia Cui

**Affiliations:** 1 Department of Life Sciences, National Cheng Kung University, Tainan, Taiwan; 2 EMBL, Institute of Oceanology, Chinese Academy of Sciences, Qingdao 266071, China; 3 Simon F. S. Li Marine Science Laboratory, School of Life Sciences, The Chinese University of Hong Kong, Shatin, Hong Kong; 4 Institute of Marine Biology and Center of Excellence for Marine Bioenvironment and Biotechnology, National Taiwan Ocean University, Keelung, Taiwan

**Keywords:** Mud shrimps, Mitochondrial genome, Gene order, Evolution, Phylogenetics

## Abstract

**Background:**

The evolutionary history and relationships of the mud shrimps (Crustacea: Decapoda: Gebiidea and Axiidea) are contentious, with previous attempts revealing mixed results. The mud shrimps were once classified in the infraorder Thalassinidea. Recent molecular phylogenetic analyses, however, suggest separation of the group into two individual infraorders, Gebiidea and Axiidea. Mitochondrial (mt) genome sequence and structure can be especially powerful in resolving higher systematic relationships that may offer new insights into the phylogeny of the mud shrimps and the other decapod infraorders, and test the hypothesis of dividing the mud shrimps into two infraorders.

**Results:**

We present the complete mitochondrial genome sequences of five mud shrimps, *Austinogebia edulis*, *Upogebia major*, *Thalassina kelanang* (Gebiidea), *Nihonotrypaea thermophilus* and *Neaxius glyptocercus* (Axiidea). All five genomes encode a standard set of 13 protein-coding genes, two ribosomal RNA genes, 22 transfer RNA genes and a putative control region. Except for *T*. *kelanang*, mud shrimp mitochondrial genomes exhibited rearrangements and novel patterns compared to the pancrustacean ground pattern. Each of the two Gebiidea species (*A*. *edulis* and *U*. *major*) and two Axiidea species (*N*. *glyptocercus* and *N*. *thermophiles*) share unique gene order specific to their infraorders and analyses further suggest these two derived gene orders have evolved independently. Phylogenetic analyses based on the concatenated nucleotide and amino acid sequences of 13 protein-coding genes indicate the possible polyphyly of mud shrimps, supporting the division of the group into two infraorders. However, the infraordinal relationships among the Gebiidea and Axiidea, and other reptants are poorly resolved. The inclusion of mt genome from more taxa, in particular the reptant infraorders Polychelida and Glypheidea is required in further analysis.

**Conclusions:**

Phylogenetic analyses on the mt genome sequences and the distinct gene orders provide further evidences for the divergence between the two mud shrimp infraorders, Gebiidea and Axiidea, corroborating previous molecular phylogeny and justifying their infraordinal status. Mitochondrial genome sequences appear to be promising markers for resolving phylogenetic issues concerning decapod crustaceans that warrant further investigations and our present study has also provided further information concerning the mt genome evolution of the Decapoda.

## Background

**Figure 1:**
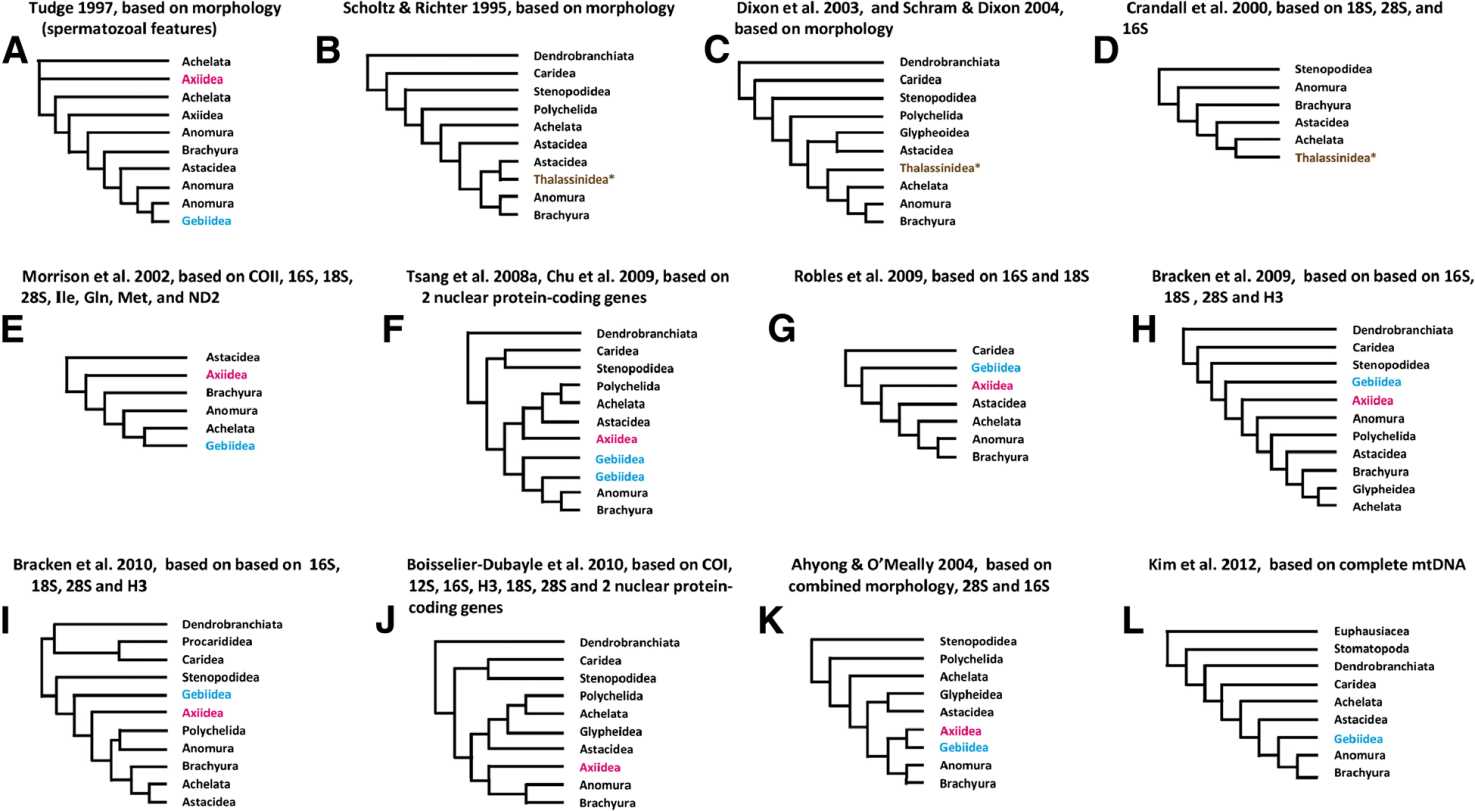
**Hypotheses of higher-level decapod relationships based on recent morphology analyses (A-C), molecular data (D-J), combined morphology and molecular data (K) and latest complete mtDNA sequence (L).** Taxa name following De Grave et al. [1] instead of original usages. *Thalassinidea refers to treat members of Gebiidea and Axiidea as forming a monophyletic group. Traditionally, lobsters (Macrura Reptantia) refer to members of Astacidea, Achelata, Glypheidea and Polychelida, while Procarididea is included in Caridea.

Decapoda is one of the most diverse groups of crustaceans, with over 15,000 extant species in 180 families [1]. Seven main groups (with the ranks of sub- or infraorder) are generally recognized in Decapoda. They are Dendrobranchiata (e.g. penaeoid shrimps and allies), Caridea (caridean shrimps), Stenopodidea (stenopodid shrimps), lobsters (Macrura Reptantia), mud shrimps or ghost shrimps (Thalassinidea or Gebiidea + Axiidea), Anomura (hermit crabs and allies) and Brachyura (true crabs). The phylogenetic relationships amongst these groups within the Decapoda and even the monophyletic status of these groups have long been debated amongst carcinologists and general consensus has yet to be reached, with recent morphological cladistic and molecular analyses still showing contrasting results (Figure 1). One the most controversial recent findings is that the mud shrimps are not monophyletic [1-8], with some of them representing the sister taxon of the other Reptantia (= non-shrimp like decapod crustaceans).

Mud shrimps have a worldwide distribution from shallow to deep waters, and more than 600 extant species have been described to date [1]. The classification scheme of mud shrimps have been in flux at all levels. They are often considered to be a monophyletic group up to the rank of infraorder, i.e., Thalassinidea [9-16]. According to different authors, these animals have been treated under Anomura [17-21], as an independent group within the Reptantia [10,11,14,22,23], or aligned with the lobsters [24]. While some authors [25-29] had long questioned the monophyly of Thalassinidea and divided it into two groups (namely Gebiidea and Axiidea), the monophyly of Thalassinidea has been supported by some morphological cladistic analyses [9,10,12,14,16], molecular data [11,30] or combined morphological and molecular analysis [15]. Nevertheless, the latest molecular analyses [3-8] mostly concur in the separation of Thalassinidea.

In most molecular analyses, partial DNA sequences are used to resolve the phylogenetic relationships of decapod crustaceans [3,11,15,22,31-34], but they are often too short to contain a sufficient amount of genetic variation for resolving higher systematics [5,35]. In the previous studies involving mud shrimps [3,4,6,7,11,15,30], the total length of partial sequences used is less than 5300 bp. The animal mitochondrial (mt) DNA is a small, extrachromosomal, and circular double-stranded DNA molecule of 12–20 kb in size, and usually contains the same set of 37 genes, including 13 protein-coding genes, two ribosomal RNA genes and 22 transfer RNA genes [36-38]. Recent advances in DNA sequencing technology have allowed rapid, cost-effective sequencing of the complete mtDNA genome. And it has become increasingly popular in studies of molecular evolution, phylogeography, and phylogenetic relationships at various taxonomic levels [38-43], mainly because of its maternal inheritance, the presence of strictly orthologous genes evolving at different rates, and lack of genetic recombination [38,44-46]. Complete mtDNA sequences provide sets of genome-level characteristics, such as gene rearrangement, which is rather conserved within some major metazoan lineages, and therefore can be especially powerful in resolving systematic relationships among higher taxa [3,40,42,47-49].

Complete mitochondrial genome sequences are now available for 37 decapod crustaceans (April, 2012; http://www.ncbi.nlm.nih.gov, with a sergesteid species *Actetes chinensis* with mitogenome reported [50] but not yet available from GenBank) that represent all the main groups. However, the latest phylogenetic reconstruction of decapod crustaceans using complete mitochondrial genome sequences [51,52] still has low resolution in most of the deep branches, notably with the status of Stenopodidea, lobsters and mud shrimps unresolved. Moreover, only a single species of mud shrimp collected from Korea, namely *Upogebia major* (De Haan, 1841) belonged to Gebiidea, has been sequenced for mitochondrial genome [53].

In this paper, we report the complete mitochondrial genomes of five thalassinidean species with representatives from both Gebiidea and Axiidea. They are *Austinogebia edulis* (Ngoc-Ho and Chan 1992), *Upogebia major* (from China) and *Thalassina kelanang* Moh and Chong, 2009 of Gebiidea, and *Nihonotrypaea thermophilus* Lin, Komai and Chan, 2007 and *Neaxius glyptocercus* (Von Martens, 1868) of Axiidea. Considering the difference in sampling location and sequence variation, we only included the mitochondrial genome of *Upogebia major* we sequenced in the analysis. The mitochondrial genome structure of these five mud shrimps were compared with those of other decapods. The gene rearrangement occurred in mud shrimps were identified. Moreover, the infraorder status of Axiidea and Gebiidea was analyzed based on all 50 malacostracan mitochondrial genomes currently available.

## Results

### Genome composition

**Figure 2:**
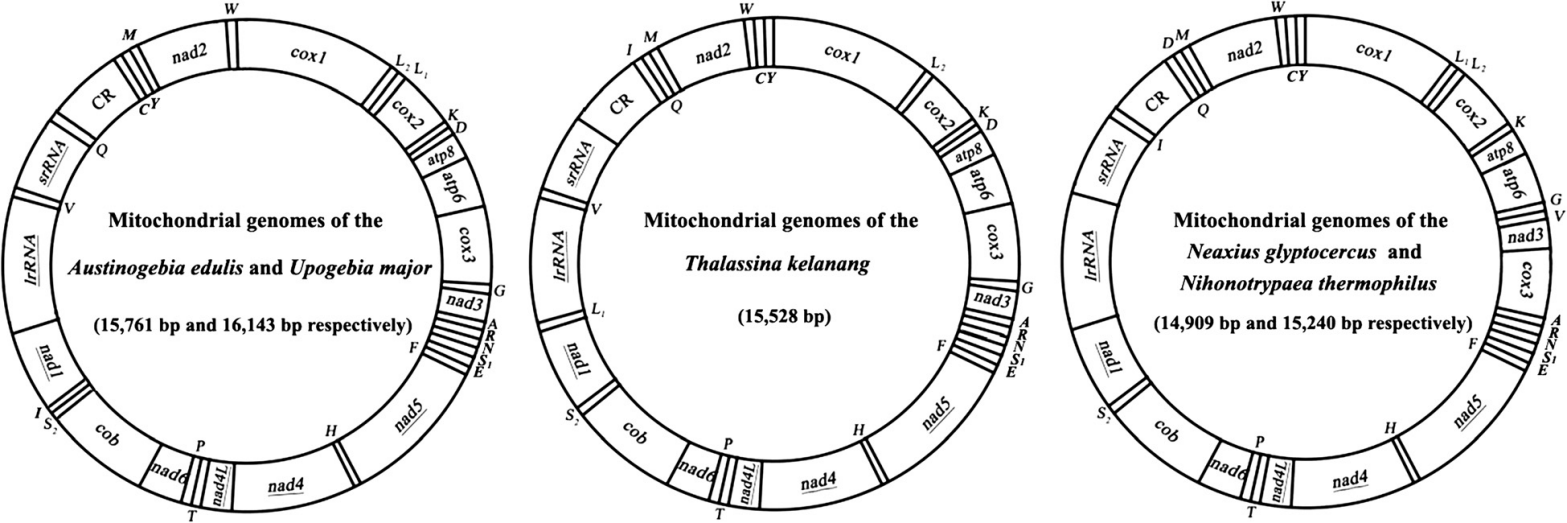
**Gene maps of the mitochondrial genomes of *Austinogebia edulis,Upogebia major,Thalassina kelanang,Neaxius glyptocercus *and *Nihonotrypaea thermophilus.*** Genes encoded on the heavy or light strands are shown outside or inside the circular gene map, respectively. The putative control region is denoted by “CR”. The tRNA genes are designated by single-letter amino acid codes except those encoding leucine and serine, *L*_*1*_, *L*_*2*_, *S*_*1*_ and *S*_*2*_ denote *tRNA*^*Leu*(*CUN*)^, *tRNA*^*Leu*(*UUR*)^, *tRNA*^*Ser*(*AGN*)^, and *tRNA*^*Ser*(*UCN*) ^genes, respectively.

The complete mtDNA sequences of *A*. *edulis*, *U*. *major*, *T*. *kelanang*, *N*. *glyptocercus* and *N*. *thermophilus* were determined to be 15,761, 16,143, 15,528, 14,909 and 15,240 bp long, respectively (Additional file 1). They all contained 13 protein-coding genes (PCGs), two ribosomal RNA genes (rRNA), 22 transfer RNA genes (tRNA) and a putative control region as in other metazoans (Figure 2; Additional files 2, 3, 4, 5, 6). The structural organizations of the five mitochondrial genomes are shown in Figure 2.

The overall A + T content of *A*. *edulis* mtDNA is 73.6%, higher than that of other decapod species except *Scylla tranquebarica* (73.8%) (see Additional file 1). The overall A + T content of *U*. *major*, *T*. *kelanang*, *N*. *glyptocercus* and *N*. *thermophilus* ranged from 66.3-70.7%, similar to other decapods (see Additional file 1). This pattern of base composition in five mud shrimps held for the protein-coding, rRNA, tRNA genes, and the control region when considered separately.

For the 13 PCGs of five mitochondrial genomes, nine protein-coding genes (*atp6*, *atp8*, *cox1*, *cox2*, *cox3*, *cob*, *nad2*, *nad3*, and *nad6*) were encoded on the H-strand, while the remaining four (*nad1*, *nad4*, *nad4L*, and *nad5*) were encoded on the L-strand (Additional files 2, 3,4, 5, 6). This transcriptional polarity is identical in all reported decapod mitochondrial genomes. Moreover, they all contained two reading frames overlapped on the same strand: *atp6* and *atp8*, *nad4* and *nad4L* each shared seven nucleotides. No notable reduction or extension of gene length as compared to other decapods was observed.

In *A*. *edulis*, *U*. *major* and *T*. *kelanang* mitochondrial genomes, *lrRNA* and *srRNA* were separated by *tRNA*^*Val*^, while the two rRNA genes in *N*. *glyptocercus* and *N*. *thermophilus* mtDNA were adjacent to each other (Figure 2). The rRNAs were both coded on L-strand. All five mitochondrial genomes had typical 22 tRNA genes, which ranged from 61 to 73 bp in length (Additional files 2, 3,4, 5, 6), and all of them (except *tRNA*^*Ser*(*AGN*)^) formed a typical cloverleaf secondary structure. The *tRNA*^*Ser*(*AGN*)^ lacked DHC arm, a feature commonly observed in metazoan mtDNAs [54].

The non-coding regions of *A*. *edulis*, *U*. *major*, *T*. *kelanang*, *N*. *glyptocercus* and *N*. *thermophilus* mtDNAs were 845, 1,188, 784, 162, 581 bp, respectively (see Additional files 2, 3,4, 5, 6). Of these regions, the largest non-coding region in each genome was assumed to be the control region (CR) with high A + T composition (Additional file 1). The mtDNA of *N*. *glyptocercus* had the shortest CR (91 bp) among decapods, and its A + T content was the lowest (59.3%) (Additional file 1). The remaining non-coding regions of the five mitochondrial genomes were considered to be intergenic spacers. Most intergenic spacers contained a few nucleotides (1–56 bp) (Additional file 2, 3,4, 5, 6). However, a relatively large spacer, 177 bp in length, was found between *srRNA* and *tRNA*^*Gl*^ in the *U*. *major* mtDNA (Figure 2 and Additional file 3). Further analyses showed that this large region had an A + T content of 89.8%, higher than that in control region (85.2%).

### Gene order

**Figure 3:**
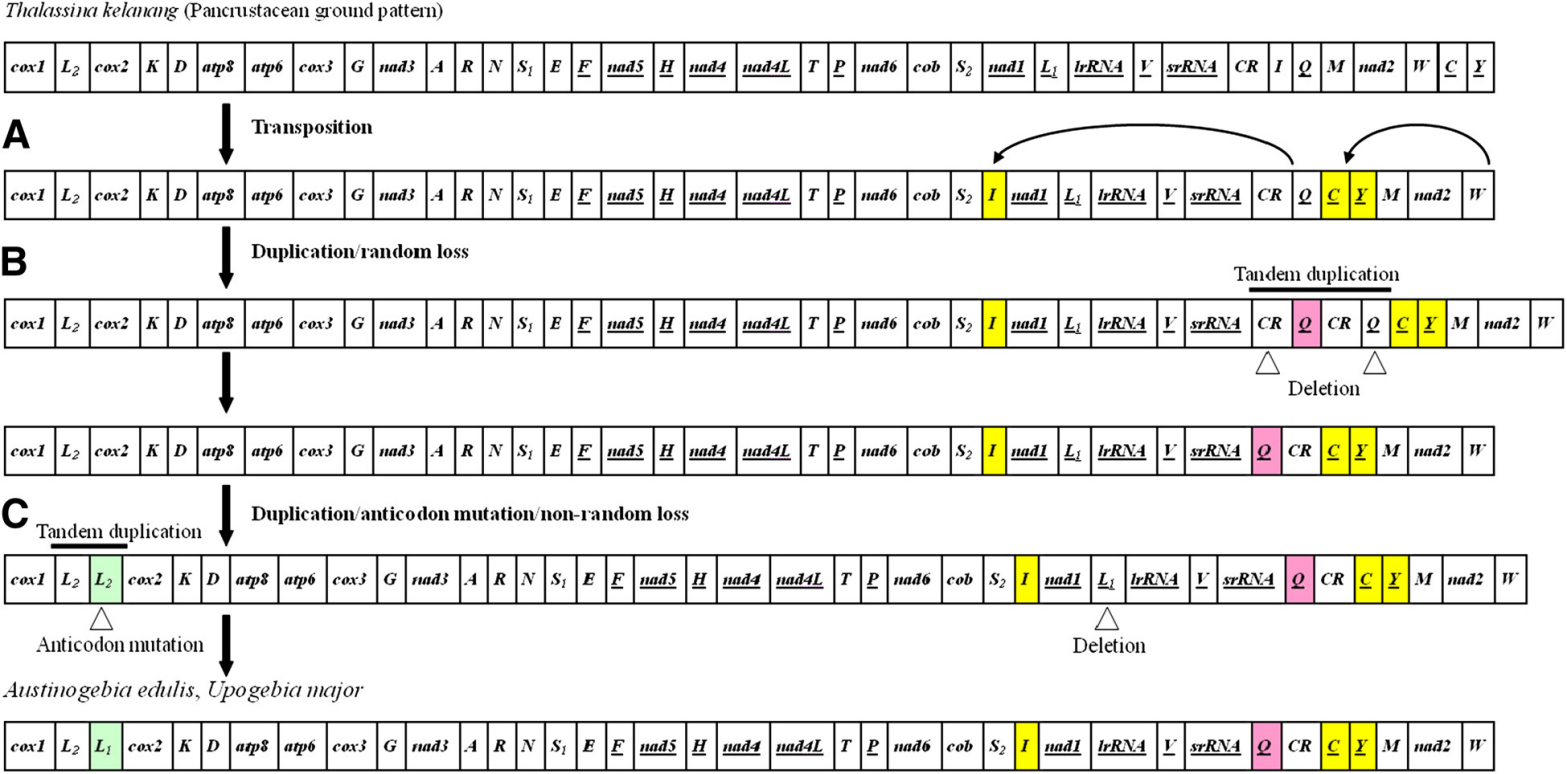
**Proposed mechanism for the mitochondrial gene arrangement of *Austinogebia edulis*, *Upogebia major *(Decapoda: Gebiidea). **Gene order of *Thalassina kelanang *is identical to that of pancrustaceans ground pattern. Arrows and shaded boxes indicate rearranged genes. Gene segments are not drawn to scale. All genes are transcribed from left to right except for those underlined that exhibit opposite transcriptional orientation.

**Figure 4:**
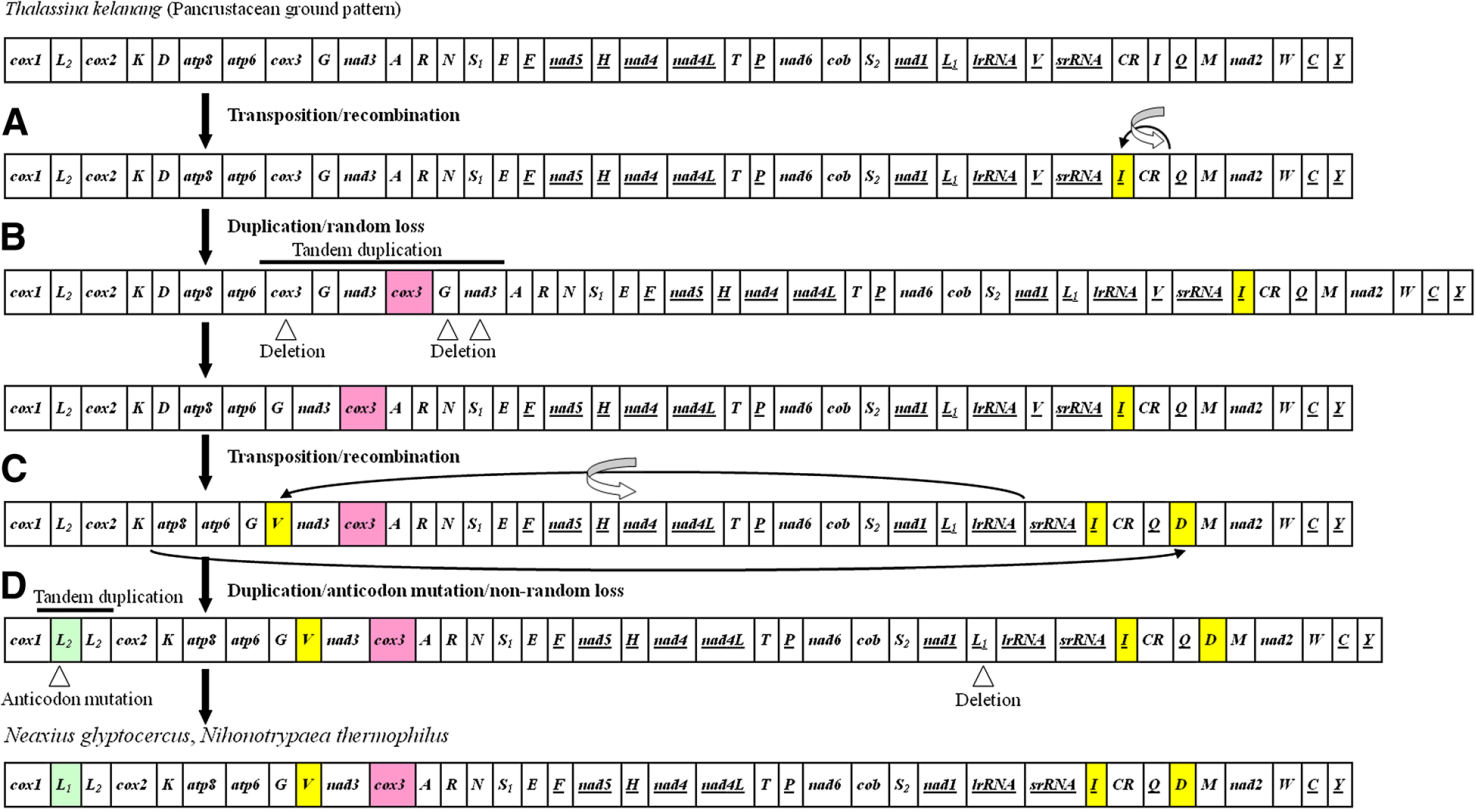
**Proposed mechanism for the mitochondrial gene arrangement of *Neaxius glyptocercus *and *Nihonotrypaea thermophilus *(Decapoda: Axiidea). **Arrows and shaded boxes indicate rearranged genes. The circling arrow indicates inversion. Gene segments are not drawn to scale. All genes are transcribed from left to right except for those underlined that exhibit opposite transcriptional orientation.

The complete genome arrangements of five mud shrimps were depicted in Figures 3 and 4. The gene order of *T*. *kelanang* mtDNA was identical to that of the pancrustacean (Crustacea + Hexapoda) ground pattern [55], while the genomic organization of four other mud shrimps showed two novel gene orders compared to other mt genomes in the MitoZoa database. Specifically, the mitochondrial genomes of *A*. *edulis* and *U*. *major*, and *N*. *glyptocercus* and *N*. *thermophilus*, shared the same gene order, respectively.

Compared with the pancrustacean ground pattern, at least five genes were rearranged in each of the mt genome of *A*. *edulis*, *U*. *major*, *N*. *glyptocercus* and *N*. *thermophilus* (Figures 3 and 4). The *tRNA*^*Leu*(*CUN*)^ (*L*_*1*_), located between *nad1* and *lrRNA* in other arthropod mtDNAs, was found between *tRNA*^*Leu*(*UUR*)^ (*L*_*2*_) and *cox2* in *A*. *edulis* and *U*. *major*, and between *cox1* and *tRNA*^*Leu*(*UUR*)^ (*L*_*1*_) in *N*. *glyptocercus* and *N*. *thermophilus*. The *tRNA*^*Ile*^ (*I*) was located between *tRNA*^*Ser*(*UCN*)^ (*S*_*2*_) and *nad1* in *A*. *edulis* and *U*. *major*, and between *srRNA* and CR in *N*. *glyptocercus* and *N*. *thermophilus*. In *A*. *edulis* and *U*. *major*, *tRNA*^*Gln*^ (*Q*) moved upstream to the putative control region, and *tRNA*^*Cys*^ (*C*) and *tRNA*^*Tyr*^ (*Y*) moved to the location between CR and *tRNA*^*Met*^ (*M*). Additionally, in *N*. *glyptocercus* and *N*. *thermophilus* mtDNAs, the tRNA gene *tRNA*^*Val*^ (*V*) changed to downstream of *tRNA*^*Gly*^ (*G*), *tRNA*^*Asp*^ (*D*) moved to upstream of *tRNA*^*Met*^ (*M*), and only one protein-coding gene *cox3* was involved in the rearrangement. The *cox3* located between *atp6* and *tRNA*^*Gly*^ (*G*) in other crustaceans moved upstream to *tRNA*^*Ala*^ (*A*) in *N*. *glyptocercus* and *N*. *thermophilus*. All these genes rearranged in the same orientation as the mitochondrial gene arrangement of pancrustacean ground pattern with the exception of *tRNA*^*Leu*(*CUN*)^ (*L*_*1*_) in the four mud shrimps, and *tRNA*^*Ile*^ (*I*) and *tRNA*^*Val*^ (*V*) in *N*. *glyptocercus* and *N*. *thermophilus*. Noticeably, the two *tRNA*^*Leu*^ sequences in each mt genome of the four mud shrimps shared significant identity with each other, and the similarity was 86% in *A*. *edulis*, 80% in *U*. *major*, 77% in *N*. *glyptocercus* and 93% in *N*. *thermophilus*.

### Phylogenetic analysis

**Figure 5:**
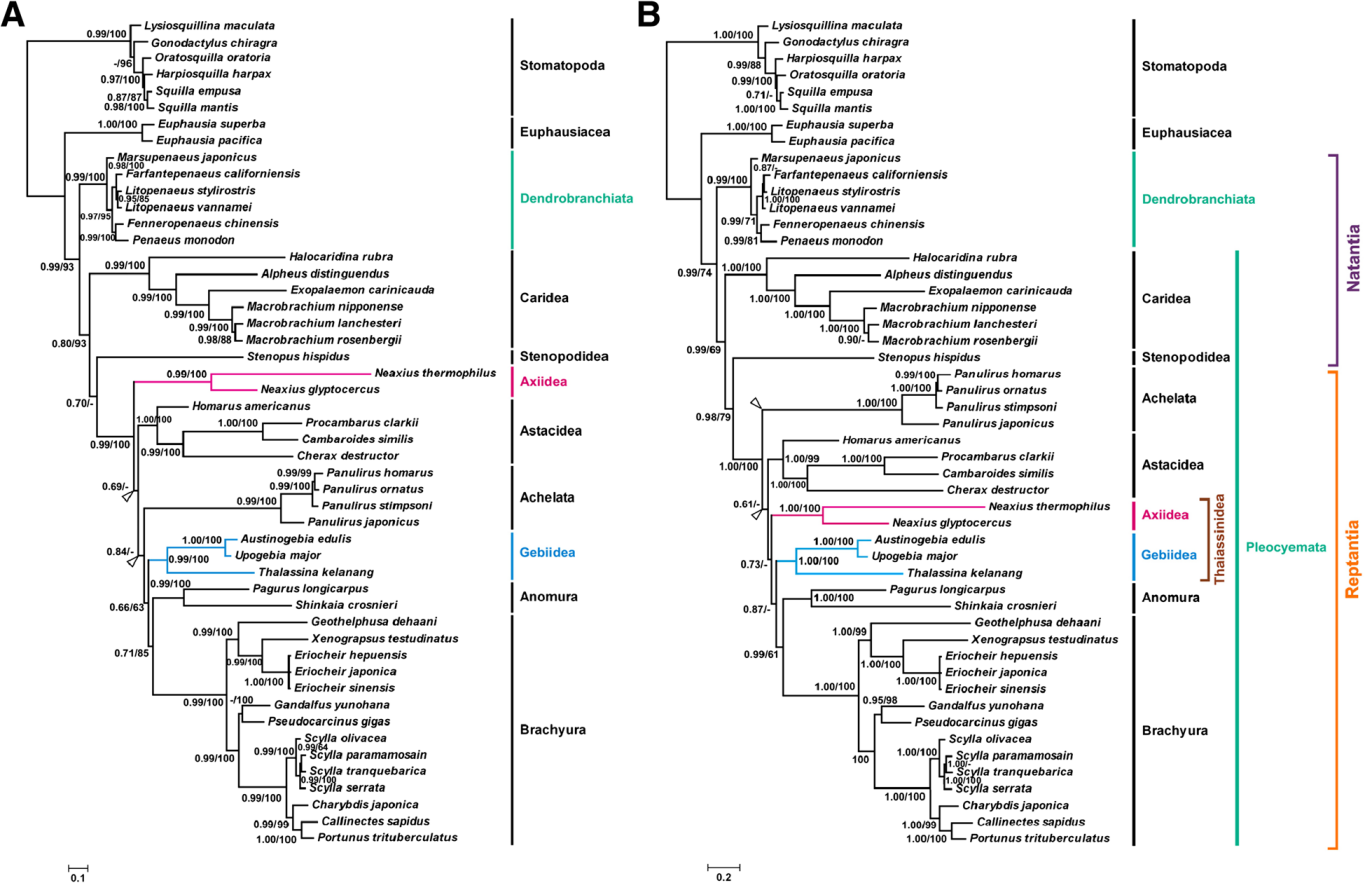
**Phylogenetic trees of the nucleotide (A) and amino acid sequence datasets (B) derived from Decapoda using Bayesian inference and maximum likelihood analysis, respectively. **Branch lengths and topologies came from Bayesian analyses. Numbers in each branch indicated Bayesian posterior probabilities (BPP)/maximum likelihood (ML) bootstrap values. The minus signs represent the bootstrap values of below 60 %. The topological incongruity between Bayesian and ML analyses is denoted by open arrowheads.

The concatenated alignments of nucleotide and amino acid data from all 13 protein-coding genes were used to investigate the phylogenetic relationships among major lineages of Decapoda. For each dataset, the BI and ML analyses generated nearly identical tree topology except for two branches denoted by open arrowheads, which strongly supported the monophyly of Decapoda (Figure 5A and B). Values of nodal support were typically congruent between the two trees. Both the nucleotide and amino acid phylogenies indicated strong support (BPP/ML bootstrap value in nucleotide phylogeny = 0.99/93, BPP/ML bootstrap value in amino acid phylogeny =0.99/74) for the separation of two suborders Dendrobranchiata and Pleocyemata in Decapoda. The placing of Caridea at the base of Pleocyemata was well supported (0.80/93, 0.99/69). The remaining natant decapod Stenopodidea was sister with Reptantia with a strong support in amino acid phylogeny (BPP/ML bootstrap value =0.98/79) but only moderate support in nucleotide phylogeny (BPP =0.70). Reptantia was strongly supported to be a monophyletic group (0.99/100, 1.00/100).

Within Repantia, the Brachyura and Anomura (i.e. Mieura) were reciprocally monophyletic (0.99/100, 1.00/100), and their sister relationship also received supported (0.71/85, 99/61). The monophyly of Thalassinidea (Gebiidea + Axiidea) was not supported in the nucleotide phylogeny or the amino acid phylogeny. Yet AU test could not reject monophyly of Thalassinidea. Gebiidea and Axiidea were both shown to be well supported clades (0.99/100, 1.00/100), with moderate support (0.66/63, 0.87/-) for the sister relationship between Gebiidea and Mieura. However, the position of Axiidea in Reptantia was incongruent between the nucleotide and amino acid trees. Similarly, there was no support for the monophyly of the lobsters (Achelata + Astacidea).

## Discussion

### Molecular features of mitochondrial genomes in mud shrimps

Features of decapod mitochondrial genomes include a high A + T content and rearranged gene structure [56-58]. These features are also apparent in the complete mtDNA sequences of four of the mud shrimps studied, i.e., *A*. *edulis*, *U*. *major*, *N*. *glyptocercus* and *N*. *thermophilus*. Together with *T*. *kelanang* mtDNA, all five mitochondrial genomes have the same gene number as other pancrustaceans (13 PCGs, 2 *rRNA*s, 22 *tRNA*s). However, the *U*. *major* mtDNA annotated by Kim et al. [53] has an extra *tRNA*^*Leu*(*CUN*)^ between *nad1* and *lrRNA*. The different annotation is due to amphibolous beginning of *lrRNA* and identification of *tRNA*^*Leu*(*CUN*)^. No anticodon and tRNA-like secondary structure of *tRNA*^*Leu*(*CUN*)^ is identified between *nad1* and *lrRNA* in the *U*. *major* mtDNA we sequenced, as in the one sequenced by Kim et al. [53].

Most variations in size in mitochondrial genomes are caused by sequences in non-coding regions [53,58]. *N*. *glyptocercus* mtDNA has the shortest control region among the decapod mtDNA published, while in *U*. *major* mtDNA, a relative large intergetic spacer (177 bp) with high A + T content is discovered. Such a large AT-rich region other than CR rarely occurs in malacostracan species and has only been reported in the stomatopods *Oratosquilla oratoria*[59] and *Squilla mantis*[60]. But the length and position of the second AT-rich region are different among the above three species. Moreover, such an AT-rich region is notably absent in the other four mud shrimps, indicating that it is not a conserved feature of thalassinidean mtDNA.

The pancrustacean ground pattern is well retained in *T*. *kelanang* mitogenome, suggesting that the ancestor of mud shrimps (or at least the Gebiidea) had a typical pancrustacean mtDNA gene order. However, the other four mud shrimps, *A*. *edulis*, *U*. *major*, *N*. *glyptocercus* and *N*. *thermophilus* have rearranged mitochondrial genomes compared to the pancrustacean ground pattern (Figure 3) [56,57,61,62]. Further searches in the MitoZoa database show that mitochondrial sequences of *A*. *edulis* and *U*. *major*, *N*. *glyptocercus* and *N*. *thermophilus* exhibit two novel genome structures, respectively. Except for *tRNA*^*Leu*(*CUN*)^, the rearranged genes of *A*. *edulis* and *U*. *major* belonged to Gebiidea occur at *tRNA*^*Ile*^—*tRNA*^*Tyr*^ junction. However, the rearranged genes of the two axiids *N*. *glyptocercus* and *N*. *thermophilus* occur more dispersedly.

### Possible mechanisms for gene rearrangement

Two major categories of mechanisms have been suggested to explain mitochondrial gene rearrangement: (1) tandem duplication followed by random or non-random deletion [63,64], and (2) non-homologous recombination [65,66]. The first mechanism may explain many or most of the observed rearrangements, while the second one has been proposed to explain gene translocation and inversion [56,67]. Combined the above mechanisms and the results from CREx (Additional files 7 and 8), the rearrangement of four mud shrimps mtDNAs can be depicted as three or four steps (Figures 3 and 4).

For *A*. *edulis* and *U*. *major* mtDNA, firstly, transposition of *tRNA*^*Ile*^ (*I*), *tRNA*^*Cys*^ (*C*) and *tRNA*^*Tyr*^ (*Y*) occurred before duplication. If this event occurred after duplication, more genes were duplicated and longer distance translocation were required (Figure 3A). Secondly, an independent duplication/random loss events occurred to account for the translocation of *tRNA*^*Gln*^ (*Q*) (Figure 3B). Thirdly, a duplication/anticodon mutation/non-random loss event [52,68] is expected to account for translocation of *tRNA*^*Leu*(*CUN*)^ (*L*_*1*_) (Figure 3C). A duplication of *tRNA*^*Leu*(*UUR*)^ (*L*_*2*_) on H-strand might have happened. One of the duplicated *tRNA*^*Leu*(*UUR*)^ (*L*_*2*_) changed into *tRNA*^*Leu*(*CUN*)^ (*L*_*1*_) by anticodon mutation. Subsequently the ancestral *tRNA*^*Leu*(*CUN*)^ (*L*_*1*_) lost the function and eventually is deleted from L-strand.

For *N*. *glyptocercus* and *N*. *thermophilus* mtDNA, transposition of *tRNA*^*Ile*^ (*I*) occurred first followed by one recombination event (Figure 4A). Then an independent duplication/random loss event occurred to account for the translocation of *cox3* (Figure 4B). This was followed by transposition of *tRNA*^*Asp*^ (D) and *tRNA*^*Val*^ (*V*) and a recombination event (Figure 4C). Finally, a duplication/anticodon mutation/non-random loss event [52,68] occurred to account for the translocation of *tRNA*^*Leu*(*CUN*)^ (*L*_*1*_) (Figure 4D).

Another possibility for the translocation of *tRNA*^*Leu*(*CUN*)^ (*L*_*1*_) predicted by CREx is based on “duplication/random loss model” [63]. This interpretation seems less likely given that there is a long distance between *tRNA*^*Leu*(*UUR*)^ (*L*_*2*_) and *tRNA*^*Leu*(*CUN*)^ (*L*_*1*_) in the ancient arrangement, as well as the presence of inversion in *tRNA*^*Leu*(*CUN*)^ (*L*_*1*_) [52]. Moreover, the sequence homology between *tRNA*^*Leu*(*UUR*)^ (*L*_*2*_) and *tRNA*^*Leu*(*CUN*)^ (*L*_*1*_) in *A*. *edulis*, *U*. *major*, *N*. *glyptocercus* and *N*. *thermophilus* is higher than any two other randomly chosen *tRNA*s. It seems to be more possible that two *tRNA*^*Leu*^ arose from duplication followed by anticodon mutation. The similar duplication/anticodon mutation events have also been reported in other crustaceans, for example in amphipods *Caprella scaura*[69] and *Gammarus duebeni*[70], and decapods *Geothelphusa dehaani*[71] and *Stenopus hispidus*[52].

Under the above models, including the random and nonrandom loss, incomplete deletion or partial retention of duplication resulted in the formation of the multiple intergenic spacers (Additional files 2, 3,4, 5, 6). These results indicate that intergenic spacers might serve as a guide in deducing the generation of gene rearrangement [56]. Moreover, the distinct rearrangement processes suggest that Gebiidea and Axiidea evolved independently from the pancrustacean ground pattern.

### Phylogenetic relationships of the major clades in Decapoda

With higher taxon samplings and the inclusion of all the major groups of decapod crustaceans, the present complete mitochondrial genomic analysis strongly supports that the Caridea is sister to the other Pleocyemata. Similar to the results of Shi et al. [52], Stenopodidae is revealed to be a sister clade of Reptantia. While this relationship is only weakly supported in Shi et al. [52], this grouping is strongly supported in our tree based on amino acid sequences.

Within the Reptantia, the sister relationship between Brachyura and Anomura (i.e. the Meiura) has always received very high support in complete mitochondrial genomic analyses [51,52,62]. The present result suggests that Gebiidea is the sister group of Meiura though only with moderate support. In general, the topology of the currently most extensive complete mitochondrial genomic tree of decapod crustaceans (particularly the one based on amino acid sequences) is most similar to the most recent mt genome analyses by Kim, Park, et al. [51], and those of Scholtz and Richter [10] and Ahyong and O’Meally [15] deduced from morphology and combined morphology and molecular (16S and 28S) data, respectively. The trees of Scholtz and Richter [10] and Ahyong and O’Meally [15] are essentially the same except for the identity of the sister clade of Thalassinidea (i.e. Gebiidea + Axiidea), which is considered to be monophyletic. Thalassinidea is sister to Mieura in Ahyong and O’Meally [15] but in Scholtz and Richter [10] it is sister to the clawed lobster Astacidea which is shown to be polyphyletic. The main difference between the present result with these two analyses is that Gebiidea and Axiidea do not make up a monophyletic group while the position of Astacidea is unresolved. The mitogenome tree of Kim et al. [50] includes only one mud shrimp species belonged to Gebiidea. Adding two more species and genera of Gebiidea in the present work reveals a similar topology of Gebiidea being sister to Mieura. However, the other mud shrimp group Axiidea does not cluster with Gebiidea.

As the results of complete mitochondrial genome sequence analysis are now rather consistent with the conclusions deduced from some morphology and partial gene sequence data, it seems to be promising in using complete mtDNA sequence to reconstruct the evolutionary history of decapod crustaceans. Nevertheless, more taxon sampling, particularly the inclusion of certain key groups such as the sergesteid shrimp (supposed to be sister to Penaeoidea in Dendrobranchiata), blind lobsters Polychelida [4], primitive cave shrimp Procarididea [7], the living fossil lobster Glypheidea [23], the enigmatic shrimp Luciferidae [72] and the various bizarre anomuran groups [73], will be necessary to achieve this goal.

## Conclusions

This study presents five complete mitochondrial genomes of mud shrimps, *Austinogebia edulis*, *Upogebia major* and *Thalassina kelanang* of Gebiidea, and *Nihonotrypaea thermophilus* and *Neaxius glyptocercus* of Axiidea. The contents of individual mt genes in these five mud shrimps are similar to that in other decapods. The *U*. *major* mt genome contain a relative large intergenic spacer with higher A + T content than that in control region. The *N*. *glyptocercus* mt genome, the shortest decapod mtDNA known, has the shortest control region. Except for *T*. *kelanang*, the other four mud shrimps have rearranged mt genomes compared to pancrustacean ground pattern. A duplication/loss (random and nonrandom) and recombination model may result in their mt gene order. The different gene arrangement process suggests the derived gene orders of Gebiidea and Axiidea might have evolved independently. Phylogenetic analyses do not support the monophyly of mud shrimps, while the positions of Gebiidea and Axiidea in Reptantia are poorly resolved.

## Methods

### Sample collection and DNA extraction

The collecting sites of the mud shrimp specimens used in the present study are *A*. *edulis* from Starfish Bay, Hong Kong, *U*. *major* from Qingdao No.1 Bathing Beach, China, *T*. *kelanang* from Kelanang Beach, Selangor, Malaysia, *N*. *glyptocercus* from Kenting, Taiwan and *N*. *thermophilus* from Kueishan Island, Taiwan. The specimens obtained were stored in 75-95% ethanol. Total genomic DNA for all species was extracted from tissues by using a DNeasy tissue kit (Qiagen) following the manufacturer’s protocol.

### PCR and sequence determination of *A*. *edulis* and *U*. *major* mitochondrial genomes

Four short fragments of the genes *cox1*, *nad5*, *lrRNA* and *cob* were first determined by PCR with the universal primer sets of LCO1490/HCO2198 [74], nad5F/nad5R [75], 16S1471/16S1472 [76] and cobF424/ cobR876 [77]. PCR products were purified using the Qiaquick Gel extraction Kit (Qiagen) and directly sequenced with ABI 3730xl DNA Analyzer.

Based on the sequences obtained above, long PCR primers were designed to amplify the entire *A*. *edulis* (AE) and *U*. *major* (UM) mitochondrial genomes in four fragments: AE/UMcox1F-AE/UMnad5R, AE/UMnad5F-AE/UMcobR, and AE/UMcobF-AE/UM16SR, AE/UM16SR-AE/UMcox1R, with the PCR products of approximate 5.5 kb, 3.7 kb, 1.9 and 4.5 kb in length, respectively (Additional file 9). PCR reactions were carried out in 25 μl reaction mixtures containing 18.8 μl of sterile distilled H_2_O, 2.5 μl of 10× LA PCR buffer II (Mg^2+^ plus, Takara), 0.5 μl of dNTP (10 mM each), 1 μl of each primer (5 μM), 0.2 μl of LA Taq polymerase (5 unit/μl, Takara), and 1 μl of DNA template (approximate 30 ng). The amplifications were performed on TaKaRa PCR Thermal Cycler Dice Model TP600 (Takara Bio Inc.) with an initial denaturation at 94° for 3 min, followed by 34 cycles of denaturation at 94° for 20 s, annealing at 50-52° for 50 s, extension at 68° for for 1 min/kb, and a final extension at 68° for 10 min. Long PCR products were purified using the Qiaquick Gel extraction Kit (Qiagen) and bidirectionally sequenced using a primer-walking strategy on ABI 3730xl DNA Analyzer.

### PCR and sequence determination of *T*. *kelanang*, *N*. *glyptocercus* and *N*. *thermophilus* mitochondrial genomes

Four partial fragments of the genes *cob*, *cox1cox2* (2 kb), *cox3*, *srRNA* were first determined by PCR with the primer sets of Cyb1/Cyb2 [78] crust-cox1f [79]/CCO2Rv1 [80], S*cox3*-F(GCCCCTTCAGTNGAAATTGG)/S*cox3*-R (ACTACATCDACRAAATGTCAATATCA), and *srRNA*-F (AAATTTAATTCAACATCGAGGTCGCAAACT)/*srRNA*-R (TTGACYGTGCRAAGGTAGCATAATAATTAG). Additional three partial mitochondrial sequences (*nad4*, *nad5* and *12S*) of *T*. *kelanang* were also determined with PCR primer sets of L11424-ND4/H11534-ND4M [81], crust-nd5f/crust-nd5r and crust-12Sf/crust-12Sr [79].

Based on the sequences obtained above, long PCR primers were designed to amplify the entire *T*. *kelanang* (TK), *N*. *glyptocercus* (NG) and *N*. *thermophilus* (NT) mitochondrial genomes in several fragments: TKbs-R/H11534-ND4M [81], TKc1s-R/TK12s-R, TKc2s-F/TKd5s-R, NGbs-F/NGrRs-R, NGc3s-F/NGbs-R, NGc1s-R/NGrRs-F, NGc2s-F/NGc2s-R, NTbs-R/NTc2s-F, and NTc1s-R/NTrRs-F, with the PCR products of approximate 2 kb, 3.7 kb, 3.7 kb, 2.3 kb, 5.6 kb, 3.6 kb, 2.5 kb, 7.5 kb and 4.2 kb in length, respectively (Additional file 10). PCR reaction and sequencing were generally the same as described in *A*. *edulis* and *U*. *major* mitochondrial genomes.

### Sequence analysis

Base calling was processed using Phred [82,83] and sequence reads were assembled using Phrap with default parameters. All assembled sequences were manually checked using CONSED to remove misassembles [84].

The locations of 13 protein-coding genes and two rRNAs were initially identified by DOGMA [85] with default settings, and refined by alignment with mitochondrial genomes of *Panulirus japonicus* (NC_004251) and *Squilla mantis* (NC_006081). A majority of tRNA genes was identified by the tRNAscan-SE 1.21 [86] in default search mode using mitochondrial/chloroplast DNA as the source and invertebrate mitochondrial genetic code for tRNA structure prediction. The remaining tRNA genes were identified by inspecting sequences for tRNA-like secondary structures and anticodons. The complete mtDNA sequences of *A*. *edulis*, *U*. *major*, *T*. *kelanang*, *N*. *glyptocercus* and *N*. *thermophilus* were deposited with GenBank under accession numbers JN897376-JN897380, respectively.

The inferred mitochondrial gene orders of the above five mud shrimps were compared with that of the 527 other arthropod species included in the MitoZoa database [87] (http://mi.caspur.it/mitozoa/index.php, Release 10, 14-Dec-2011). The genome rearrangement steps were predicted by algorithms implanted in CREx server [88] together with gene rearrangement models reported in previous arthropod mitochondrial genomes [56].

### Phylogenetic analysis

Along with the complete mtDNA sequences from *A*. *edulis*, *U*. *major*, *T*. *kelanang*, *N*. *glyptocercus* and *N*. *thermophilus*, all currently available 37 decapod complete mitochondrial sequences (see Additional file 1) were used in phylogenetic analysis. The six stomatopods *Gonodactylus chiragra* (GenBank accession number: NC_007442), *Harpiosquilla harpax* (NC_006916), *Lysiosquilla harpax* (NC_007443), *Oratosquilla oratoria* (NC_014342), *Squilla empusa* (NC_007444) and *Squilla mantis* (NC_006081), and two euphausians *Euphausia pacifica* (NC_016184) and *Euphausia superba* (EU583500) served as outgroups. Both nucleotides and amino acids of 13 protein-coding genes were subjected to concatenated alignments using ClustalX 1.83 with the default settings [89]. For the nucleotides, we omitted the third codon position before alignment, according to the result of a saturation analysis [90] by DAMBE version 5.0.32 [91]. The final nucleotide and amino acid datasets consisted of 7,577 and 3,781 sites, respectively. Phylogenetic trees were built by two approaches including Bayesian inference (BI) analysis using Phylobayes 3.3b [92] and maximum-likelihood (ML) analysis using RaxML 7.0.4 [93].

For the nucleotide dataset, the model GTR + I + G was selected by JMODELTEST 0.1.1 [94]. The model MtRev + I + G + F was chosen as the best-fit model for the amino acid dataset by ProtTest version 2.4 [95]. According to preliminary analysis, the categories model with GTR (CAT-GTR) and CAT-Poisson models [96,97] fit the data best and were used for BI and ML analysis of the nucleotide and amino acid data, respectively. For BI analysis, two independent MCMC chains were run simultaneously to determine whether the searching reached stabilization, and were stopped when all chains converged (maxdiff less than 0.1). For ML analysis, 1000 bootstraps were used to estimate the node reliability. Topology testing was performed using Consel [98] for the approximately unbiased (AU) test [99].

## Abbreviations

*atp6* and *8*: ATPase subunit 6 and 8; bp: Base pair (s); BI: Bayesian inference; BP: Bootstrap; *cox1*-*3*: Cytochrome *c* oxidase subunits I-III; *cob*: Cytochrome b; *lrRNA*: 16S ribosomal RNA; ML: Maximum likelihood; Mt: Mitochondrial; mtDNA: Mitochondrial DNA; nt: Nucleotide (s); *nad1*-*6* and *4 L*: NADH dehydrogenase subunits 1–6 and 4 L; ORF: Open reading frame; PCG: Protein coding gene; PCR: Polymerase chain reaction; BPP: Bayesian posterior probabilities; rRNA: Ribosomal RNA; *srRNA*: 12S ribosomal RNA; tRNA: Transfer RNA; tRNA gene: trnX (where X is replaced by single letter amino acid code of the corresponding amino acid).

## Competing interests

The authors declare that they have no competing interests.

## Authors’ contributions

TYC, KHC and ZC contributed to the conception and design of the study. FJL and YL performed the laboratory works of *A*. *edulis*, *T*. *kelanang*, *N*. *glyptocercus* and *N*. *thermophilus*. ZS and RL conducted the work on *U*. *major*. YL and LMT performed bioinformatics analyses of nucleotide and protein sequences. FJL and YL cooperated with the writing of the manuscript. ZC supervised the study and wrote the final draft of the manuscript. All authors read and approved the final manuscript.

## Supplementary Material

Additional file 1 Genomic characteristics of decapod mitochondrial genomes. Click here for file

Additional file 2 **Location of genes in the mitochondrial genome of *Austinogebia edulis*.** Click here for file

Additional file 3 **Location of genes in the mitochondrial genome of *Upogebia major*.** Click here for file

Additional file 4 **Location of genes in the mitochondrial genome of *Thalassina kelanang*.** Click here for file

Additional file 5 **Location of genes in the mitochondrial genome of *Neaxius glyptocercus*.** Click here for file

Additional file 6 **Location of genes in the mitochondrial genome of *Nihonotrypaea thermophilus*.** Click here for file

Additional file 7 **Mitochondrial gene order rearrangement scenario of *Austinogebia edulis*, *Upogebia major *(Decapoda: Gebiidea) inferred by CREx. **The elements in the blue shaded boxes are lost in the second copy, therefore the remaining copies are moved to the front. The elements in the red shaded boxes are lost in the first copy, therefore the remaining copies are moved to the back. Click here for file

Additional file 8 **Mitochondrial gene order rearrangement scenario of *Neaxius glyptocercus *and *Nihonotrypaea thermophilus *(Decapoda: Axiidea) inferred by CREx. **The elements in the blue shaded boxes are lost in the second copy, therefore the remaining copies are moved to the front. The elements in the red shaded boxes are lost in the first copy, therefore the remaining copies are moved to the back. Click here for file

Additional file 9 **Specific primers used in amplification of *Austinogebia edulis *and *Upogebia major *mitochondrial genomes.** Click here for file

Additional file 10 **Specific primers used to amplification of the fragments which covered the gaps after sequencing*Thalassina kelanang*, *Neaxius glyptocercus *and *Nihonotrypaea thermophilus *mitochondrial genomes.** Click here for file
