# Supplementary material for: Evolution and phylogeny of the mud shrimps (Crustacea: Decapoda) revealed from complete mitochondrial genomes
Source: BMC Genomics. 2012 Nov 16;13:631. doi: 10.1186/1471-2164-13-631 (PMC3533576; doi:10.1186/1471-2164-13-631)
Supplement: Additional file 1 — Genomic characteristics of decapod mitochondrial genomes. [file 1471-2164-13-631-S1.doc]

**Additional File 1** Genomic characteristics of decapod mitochondrial genomes

| Species | Accession No | L-strand | | Protein-coding genes | | | lrRNA genes | | srRNA genes | | tRNA gens | | Purative control region | |
| --- | --- | --- | --- | --- | --- | --- | --- | --- | --- | --- | --- | --- | --- | --- |
|  |  | Length  (bp) | A+T  (%) | Length  (aa) | A+T (%) | | Length  (bp) | A+T  (%) | Length  (bp) | A+T  (%) | Length  (bp) | A+T  (%) | Length  (bp) | A+T  (%) |
|  |  |  |  |  | all | 3rd |  |  |  |  |  |  |  |  |
| *Callinectes sapidus* | NC_006281 | 16263 | 69.1 | 3712 | 67.0 | 76.5 | 1323 | 71.8 | 785 | 70.3 | 1463 | 71.6 | 1435 | 78.2 |
| *Charybdis japonica* | NC_013246 | 15738 | 69.2 | 3712 | 67.8 | 78.0 | 1317 | 74.2 | 834 | 70.3 | 1458 | 70.9 | 863 | 74.7 |
| *Portunus trituberculatus* | NC_005037 | 16026 | 70.2 | 3715 | 68.8 | 81.4 | 1332 | 73.8 | 840 | 70.1 | 1468 | 72.0 | 1104 | 76.3 |
| *Scylla olivacea* | NC_012569 | 15723 | 69.4 | 3715 | 67.3 | 76.3 | 1337 | 74.4 | 852 | 72.4 | 1482 | 72.3 | 778 | 79.0 |
| *Scylla paramamosain* | NC_012572 | 15825 | 73.0 | 3715 | 70.8 | 84.4 | 1340 | 77.4 | 870 | 75.9 | 1482 | 74.5 | 833 | 86.2 |
| *Scylla serrata* | NC_012565 | 15775 | 72.5 | 3715 | 70.7 | 84.1 | 1355 | 76.5 | 874 | 74.8 | 1484 | 74.1 | 786 | 84.5 |
| *Scylla tranquebarica* | NC_012567 | 15833 | 73.8 | 3716 | 72.0 | 87.0 | 1339 | 77.1 | 869 | 75.9 | 1486 | 74.4 | 854 | 86.5 |
| *Pseudocarcinus gigas* | NC_006891 | 15515 | 70.5 | 3734 | 68.9 | 79.6 | 1324 | 74.8 | 821 | 73.8 | 1460 | 73.2 | 593 | 80.3 |
| *Gandalfus yunohana* | NC_013713 | 15567 | 69.9 | 3716 | 68.4 | 78.5 | 1356 | 75.7 | 842 | 74.2 | 1473 | 72 | 625 | 72.8 |
| *Eriocheir hepuensis* | NC_011598 | 16335 | 71.5 | 3718 | 69.0 | 79.5 | 1310 | 77.6 | 892 | 77.1 | 1464 | 72.5 | 873 | 80.8 |
| *Eriocheir japonica* | NC_011597 | 16352 | 71.6 | 3718 | 69.4 | 80.7 | 1310 | 77.5 | 892 | 76.7 | 1462 | 72.0 | 896 | 80.5 |
| *Eriocheir sinensis* | NC_006992 | 16354 | 71.7 | 3718 | 68.9 | 79.5 | 1311 | 77.4 | 899 | 76.6 | 1473 | 72.4 | 896 | 83.1 |
| *Xenograpsus testudinatus* | NC_013480 | 15798 | 73.9 | 3718 | 71.8 | 86.1 | 1301 | 78.6 | 883 | 78.7 | 1478 | 74.5 | 523 | 83.9 |
| *Geothelphusadehaani* | NC_007379 | 18197 | 74.9 | 3711 | 71.5 | 83.4 | 1315 | 77.1 | 821 | 76.4 | 1519 | 75.8 | 514 | 87.2 |
| *Pagurus longicarpusa* ^a^ | NC_003058 | / | / | 3698 | 69.6 | 84.0 | 1303 | 77.1 | 789 | 77.2 | 1458 | 74.1 | / | / |
| *Shinkaia crosnierib* | NC_011013 | 15182 | 72.9 | 3698 | 71.0 | 82.3 | 1331 | 77.8 | 811 | 78.1 | 1194 | 73.7 | 327 | 83.5 |
| *Cherax destructor* | NC_011243 | 15895 | 62.4 | 3705 | 60.0 | 59.6 | 1302 | 67.9 | 917 | 68.3 | 1436 | 70.7 | 977 | 65.8 |
| *Homarus americanus* | NC_015607 | 16432 | 69.5 | 3713 | 67.8 | 81.8 | 1340 | 73.7 | 843 | 74.3 | 1468 | 70.4 | 1488 | 74.0 |
| *Cambaroides similis* | NC_016925 | 16220 | 71.6 | 3707 | 69.8 | 78.8 | 1264 | 75.6 | 787 | 75.1 | 1412 | 74.0 | 1455 | 77.7 |
| *Procambarus clarkii* | NC_016926 | 15928 | 72.9 | 3708 | 71.2 | 81.6 | 1262 | 76.5 | 786 | 76.5 | 1422 | 73.8 | 1188 | 80.9 |
| *Panulirus stimpsoni* | NC_014339 | 15677 | 65.6 | 3715 | 63.8 | 69.3 | 1347 | 69.3 | 859 | 68.5 | 1470 | 69.0 | 744 | 74.3 |
| *Panulirus japonicus* | NC_004251 | 15717 | 64.5 | 3715 | 62.6 | 66.8 | 1355 | 69.2 | 855 | 67.1 | 1484 | 68.9 | 786 | 70.6 |
| *Panulirus ornatus* | NC_014854 | 16105 | 66.7 | 3716 | 65.1 | 71.9 | 1363 | 72.4 | 856 | 69.0 | 1468 | 69.1 | 1176 | 69.4 |
| *Panulirus homarus* | NC_016015 | 15665 | 67.1 | 3715 | 65.2 | 72.2 | 1347 | 73.5 | 859 | 69.7 | 1452 | 69.5 | 738 | 74.7 |
| *Exopalaemon carinicauda* | NC_012566 | 15730 | 63.6 | 3696 | 60.5 | 66.5 | 1296 | 71.8 | 846 | 71.3 | 1446 | 65.8 | 886 | 79.7 |
| *Macrobrachium nipponense* | NC_015073 | 15806 | 66.0 | 3698 | 64.2 | 73.9 | 1305 | 68.8 | 852 | 69.0 | 1450 | 65.7 | 950 | 79.9 |
| *Alpheus distinguendus* | NC_014883 | 15700 | 60.2 | 3705 | 57.8 | 58.1 | 1299 | 64.8 | 872 | 60.3 | 1451 | 63.8 | 890 | 76.6 |
| *Halocaridina rubra* | NC_008413 | 16065 | 63.2 | 3701 | 60.2 | 62.8 | 1351 | 68.2 | 872 | 68.8 | 1471 | 67.8 | 1020 | 78.4 |
| *Macrobrachium rosenbergii* | NC_006880 | 15772 | 62.3 | 3708 | 60.1 | 63.9 | 1305 | 66 | 852 | 66 | 1449 | 64.7 | 931 | 75.7 |
| *Macrobrachium lanchesteri* | NC_012217 | 15694 | 67.1 | 3698 | 65.2 | 75.1 | 1305 | 71.6 | 849 | 70.3 | 1450 | 66.4 | 861 | 82.7 |
| *Penaeus monodon* | NC_002184 | 15984 | 70.6 | 3716 | 69.3 | 83.7 | 1365 | 74.9 | 852 | 71.6 | 1494 | 68 | 991 | 81.5 |
| *Marsupenaeus japonicus* | NC_007010 | 15968 | 66.5 | 3712 | 64.7 | 73.9 | 1367 | 70.5 | 853 | 67.9 | 1483 | 64 | 992 | 82.5 |
| *Fenneropenaeus chinensis* | NC_009679 | 16004 | 68.9 | 3710 | 67.5 | 80.7 | 1367 | 72.7 | 852 | 69.9 | 1501 | 65.9 | 997 | 82.3 |
| *Litopenaeus vannamei* | NC_009626 | 15990 | 67.7 | 3709 | 66.0 | 76.9 | 1369 | 71.7 | 856 | 69.4 | 1493 | 65.2 | 995 | 82.9 |
| *Farfantepenaeus californiensis* | NC_012738 | 15975 | 67.0 | 3709 | 65.3 | 75.3 | 1364 | 72.0 | 852 | 68.5 | 1490 | 64.9 | 990 | 81.0 |
| *Litopenaeus stylirostris* | NC_012060 | 15988 | 68.6 | 3710 | 67.4 | 80.9 | 1364 | 72.0 | 852 | 69.7 | 1492 | 65.0 | 999 | 80.7 |
| *Stenopus hispidus* | JN399096 | 15528 | 70.6 | 3702 | 68.6 | 82.5 | 1353 | 77.2 | 822 | 74.6 | 1505 | 71.6 | 566 | 78.6 |
| ***Austinogebia edulis* ^b^** | **JN897376** | **15761** | **73.6** | **3711** | **72.1** | **88.5** | **1410** | **76.4** | **899** | **78.2** | **1465** | **72.7** | **764** | **85.2** |
| ***Upogebia major* ^b^** | **JN897377** | **16143** | **70.7** | **3711** | **68.1** | **80.7** | **1444** | **75.7** | **893** | **75.7** | **1484** | **70.4** | **784** | **85.2** |
| ***Thalassina kelanang* ^b^** | **JN897378** | **15528** | **66.3** | **3712** | **64.1** | **70.3** | **1317** | **71.1** | **842** | **72.9** | **1440** | **68.8** | **713** | **77.4** |
| ***Neaxius glyptocercus* ^b^** | **JN897379** | **14909** | **67.4** | **3704** | **66.5** | **77.3** | **1397** | **70.8** | **797** | **68.1** | **1434** | **69.7** | **91** | **59.3** |
| ***Nihonotrypaea thermophilus* ^b^** | **JN897380** | **15240** | **69.6** | **3693** | **68.1** | **80.3** | **1357** | **75.4** | **782** | **72.8** | **1443** | **72.4** | **546** | **70.9** |

^a^ Incomplete mtDNA sequence.

^b^ Results of *Austinogebia edulis*, *Upogebia major*, *Thalassina kelanang*, *Neaxius glyptocercus* and *Nihonotrypaea thermophilus* in this study are given in bold.
