# Supplementary material for: Evolution and phylogeny of the mud shrimps (Crustacea: Decapoda) revealed from complete mitochondrial genomes
Source: BMC Genomics. 2012 Nov 16;13:631. doi: 10.1186/1471-2164-13-631 (PMC3533576; doi:10.1186/1471-2164-13-631)
Supplement: Additional file 2 — Location of genes in the mitochondrial genome of Austinogebia edulis. [file 1471-2164-13-631-S2.doc]

***Additional File 2*** *Location of genes in the mitochondrial genome of Austinogebia edulis*

| Gene | Position | | Size | | Codon | | Intergenic nucleotides^b^ | Strand |
| --- | --- | --- | --- | --- | --- | --- | --- | --- |
|  | From | To | Nucleotide | Amino acid | Start | Stop^a^ |  |  |
| *cox1* | 1 | 1542 | 1542 | 513 | ATA | TAA | 0 | H |
| *tRNA^Leu(UUR)^* | 1543 | 1608 | 66 |  |  |  | 3 | H |
| *tRNA^Leu(CUN)^* | 1612 | 1678 | 67 |  |  |  | 4 | H |
| *cox2* | 1683 | 2367 | 685 | 228 | ATG | Taa | 0 | H |
| *tRNA^Lys^* | 2368 | 2435 | 68 |  |  |  | 0 | H |
| *tRNA^Asp^* | 2436 | 2501 | 66 |  |  |  | 0 | H |
| *atp8* | 2502 | 2660 | 159 | 52 | ATG | TAA | -7 | H |
| *atp6* | 2654 | 3328 | 675 | 224 | ATG | TAA | -1 | H |
| *cox3* | 3328 | 4117 | 790 | 263 | ATG | Taa | 0 | H |
| *tRNA^Gly^* | 4118 | 4182 | 65 |  |  |  | 0 | H |
| *nad3* | 4183 | 4536 | 354 | 117 | ATT | TAA | -2 | H |
| *tRNA^Ala^* | 4535 | 4600 | 66 |  |  |  | 0 | H |
| *tRNA^Arg^* | 4601 | 4663 | 63 |  |  |  | -1 | H |
| *tRNA^Asn^* | 4663 | 4730 | 68 |  |  |  | -1 | H |
| *tRNA^Ser(AGN)^* | 4730 | 4798 | 69 |  |  |  | 1 | H |
| *tRNA^Glu^* | 4800 | 4864 | 65 |  |  |  | 0 | H |
| *tRNA^Phe^* | 4865 | 4930 | 66 |  |  |  | -1 | L |
| *nad5* | 4930 | 6660 | 1731 | 576 | ATG | TAA | 0 | L |
| *tRNA^His^* | 6661 | 6724 | 64 |  |  |  | -1 | L |
| *nad4* | 6724 | 8064 | 1341 | 446 | ATG | TAG | -7 | L |
| *nad4L* | 8058 | 8357 | 300 | 99 | ATG | TAA | 1 | L |
| *tRNA^Thr^* | 8359 | 8424 | 66 |  |  |  | 0 | H |
| *tRNA^Pro^* | 8425 | 8490 | 66 |  |  |  | 2 | L |
| *nad6* | 8493 | 9002 | 510 | 169 | ATT | TAA | -1 | H |
| *cob* | 9002 | 10136 | 1135 | 378 | ATG | Taa | 0 | H |
| *tRNA^Ser(UCN)^* | 10137 | 10204 | 68 |  |  |  | 0 | H |
| *tRNA^Ile^* | 10205 | 10269 | 65 |  |  |  | 11 | H |
| *nad1* | 10281 | 11222 | 942 | 313 | ATA | TAA | 0 | L |
| *lrRNA* | 11223 | 12632 | 1410 |  |  |  | 0 | L |
| *tRNA^Val^* | 12633 | 12701 | 69 |  |  |  | 0 | L |
| *srRNA* | 12702 | 13600 | 899 |  |  |  | 0 | L |
| *tRNA^Gln^* | 13601 | 13668 | 68 |  |  |  | 0 | L |
| *nCR* | 13669 | 14432 | 764 |  |  |  | 0 |  |
| *tRNA^Cys^* | 14433 | 14499 | 67 |  |  |  | 3 | L |
| *tRNA^Tyr^* | 14503 | 14568 | 66 |  |  |  | 56 | L |
| *tRNA^Met^* | 14625 | 14693 | 69 |  |  |  | 0 | H |
| *nad2* | 14694 | 15695 | 1002 | 333 | ATT | TAA | -2 | H |
| *tRNA^Trp^* | 15694 | 15761 | 68 |  |  |  | 0 | H |

^a^ TAa and Taa represent incomplete stop codons.

^b^ Numbers correspond to the nucleotides separating adjacent genes. Negative numbers indicate overlapping nucleotides.
