# Supplementary material for: Evolution and phylogeny of the mud shrimps (Crustacea: Decapoda) revealed from complete mitochondrial genomes
Source: BMC Genomics. 2012 Nov 16;13:631. doi: 10.1186/1471-2164-13-631 (PMC3533576; doi:10.1186/1471-2164-13-631)
Supplement: Additional file 3 — Location of genes in the mitochondrial genome of Upogebia major. [file 1471-2164-13-631-S3.doc]

**Additional File 3** Location of genes in the mitochondrial genome of *Upogebia major*

| Gene | Position | | Size | | Codon | | Intergenic nucleotides^b^ | Strand |
| --- | --- | --- | --- | --- | --- | --- | --- | --- |
|  | From | To | Nucleotide | Amino acid | Start | Stop^a^ |  |  |
| *cox1* | 1 | 1539 | 1539 | 512 | ACG | TAA | -1 | H |
| *tRNA^Leu(UUR)^* | 1539 | 1606 | 68 |  |  |  | 1 | H |
| *tRNA^Leu(CUN)^* | 1608 | 1672 | 65 |  |  |  | 7 | H |
| *cox2* | 1680 | 2364 | 685 | 228 | ATG | Taa | 0 | H |
| *tRNA^Lys^* | 2365 | 2432 | 68 |  |  |  | -1 | H |
| *tRNA^Asp^* | 2432 | 2497 | 66 |  |  |  | 0 | H |
| *atp8* | 2498 | 2656 | 159 | 52 | ATG | TAA | -7 | H |
| *atp6* | 2650 | 3324 | 675 | 224 | ATG | TAA | -1 | H |
| *cox3* | 3324 | 4113 | 790 | 263 | ATG | Taa | 0 | H |
| *tRNA^Gly^* | 4114 | 4178 | 65 |  |  |  | 0 | H |
| *nad3* | 4179 | 4532 | 354 | 117 | ATT | TAA | -2 | H |
| *tRNA^Ala^* | 4531 | 4595 | 65 |  |  |  | 0 | H |
| *tRNA^Arg^* | 4596 | 4658 | 63 |  |  |  | -1 | H |
| *tRNA^Asn^* | 4658 | 4728 | 71 |  |  |  | -1 | H |
| *tRNA^Ser(AGN)^* | 4728 | 4796 | 69 |  |  |  | 0 | H |
| *tRNA^Glu^* | 4797 | 4864 | 68 |  |  |  | 0 | H |
| *tRNA^Phe^* | 4865 | 4931 | 67 |  |  |  | -3 | L |
| *nad5* | 4929 | 6660 | 1732 | 577 | ATG | Taa | 0 | L |
| *tRNA^His^* | 6661 | 6725 | 65 |  |  |  | -1 | L |
| *nad4* | 6725 | 8065 | 1341 | 446 | ATG | TAG | -7 | L |
| *nad4L* | 8059 | 8358 | 300 | 99 | ATG | TAA | 1 | L |
| *tRNA^Thr^* | 8360 | 8427 | 68 |  |  |  | 0 | H |
| *tRNA^Pro^* | 8428 | 8493 | 66 |  |  |  | 8 | L |
| *nad6* | 8502 | 9011 | 510 | 169 | ATT | TAA | -1 | H |
| *cob* | 9011 | 10145 | 1135 | 378 | ATG | Taa | 0 | H |
| *tRNA^Ser(UCN)^* | 10146 | 10213 | 68 |  |  |  | 2 | H |
| *tRNA^Ile^* | 10216 | 10283 | 68 |  |  |  | 18 | H |
| *nad1* | 10302 | 11243 | 942 | 313 | ATG | TAG | 0 | L |
| *lrRNA* | 11244 | 12687 | 1444 |  |  |  | 0 | L |
| *tRNA^Val^* | 12688 | 12757 | 70 |  |  |  | 0 | L |
| *srRNA* | 12758 | 13650 | 893 |  |  |  | 177 | L |
| *tRNA^Gln^* | 13828 | 13896 | 69 |  |  |  | 0 | L |
| *CR* | 13897 | 14816 | 920 |  |  |  | 0 |  |
| *tRNA^Cys^* | 14817 | 14882 | 66 |  |  |  | 0 | L |
| *tRNA^Tyr^* | 14883 | 14951 | 69 |  |  |  | 50 | L |
| *tRNA^Met^* | 15002 | 15074 | 73 |  |  |  | -2 | H |
| *nad2* | 15073 | 16072 | 1000 | 333 | ATT | Taa | 1 | H |
| *tRNA^Trp^* | 16074 | 16140 | 67 |  |  |  | 3 | H |

^a^ TAa and Taa represent incomplete stop codons.

^b^ Numbers correspond to the nucleotides separating adjacent genes. Negative numbers indicate overlapping nucleotides.
