# Supplementary material for: Evolution and phylogeny of the mud shrimps (Crustacea: Decapoda) revealed from complete mitochondrial genomes
Source: BMC Genomics. 2012 Nov 16;13:631. doi: 10.1186/1471-2164-13-631 (PMC3533576; doi:10.1186/1471-2164-13-631)
Supplement: Additional file 4 — Location of genes in the mitochondrial genome of Thalassina kelanang. [file 1471-2164-13-631-S4.doc]

***Additional File 4*** *Location of genes in the mitochondrial genome of Thalassina kelanang*

| Gene | Position | | Size | | Codon | | Intergenic nucleotides^b^ | Strand |
| --- | --- | --- | --- | --- | --- | --- | --- | --- |
|  | From | To | Nucleotide | Amino acid | Start | Stop^a^ |  |  |
| *cox1* | 1 | 1536 | 1536 | 511 | ATT | TAA | 1 | H |
| *tRNA^Leu(UUR)^* | 1538 | 1601 | 64 |  |  |  | 3 | H |
| *cox2* | 1605 | 2289 | 685 | 228 | ATG | Taa | 0 | H |
| *tRNA^Lys^* | 2290 | 2356 | 67 |  |  |  | 0 | H |
| *tRNA^Asp^* | 2357 | 2422 | 66 |  |  |  | 0 | H |
| *atp8* | 2423 | 2581 | 159 | 52 | ATG | TAA | -7 | H |
| *atp6* | 2575 | 3252 | 678 | 225 | ATG | TAA | -1 | H |
| *cox3* | 3252 | 4041 | 790 | 263 | ATG | Taa | 0 | H |
| *tRNA^Gly^* | 4042 | 4106 | 65 |  |  |  | 0 | H |
| *nad3* | 4107 | 4458 | 352 | 117 | ATT | Taa | 0 | H |
| *tRNA^Ala^* | 4469 | 4522 | 64 |  |  |  | -2 | H |
| *tRNA^Arg^* | 4521 | 4584 | 64 |  |  |  | -1 | H |
| *tRNA^Asn^* | 4584 | 4649 | 66 |  |  |  | 0 | H |
| *tRNA^Ser(UCN)^* | 4650 | 4716 | 67 |  |  |  | 7 | H |
| *tRNA^Glu^* | 4724 | 4787 | 64 |  |  |  | 2 | H |
| *tRNA^Phe^* | 4790 | 4852 | 63 |  |  |  | -1 | L |
| *nad5* | 4852 | 6582 | 1731 | 576 | ATT | TAA | 0 | L |
| *tRNA^His^* | 6583 | 6646 | 64 |  |  |  | -1 | L |
| *nad4* | 6646 | 7985 | 1340 | 446 | ATG | TAa | -7 | L |
| *nad4L* | 7979 | 8281 | 303 | 100 | ATG | TAA | 1 | L |
| *tRNA^Thr^* | 8283 | 8346 | 64 |  |  |  | 0 | H |
| *tRNA^Pro^* | 8347 | 8410 | 64 |  |  |  | 2 | L |
| *nad6* | 8413 | 8925 | 513 | 170 | ATT | TAA | 2 | H |
| *cob* | 8928 | 10064 | 1137 | 378 | ATG | TAG | -2 | H |
| *tRNA^Ser(UCN)^* | 10063 | 10127 | 65 |  |  |  | 14 | H |
| *nad1* | 10142 | 11089 | 948 | 315 | GTG | TAA | 20 | L |
| *tRNA^Leu(CUN)^* | 11110 | 11174 | 65 |  |  |  | 0 | L |
| *lrRNA* | 11175 | 12491 | 1317 |  |  |  | 0 | L |
| *tRNA^Val^* | 12492 | 12562 | 71 |  |  |  | 0 | L |
| *srRNA* | 12563 | 13404 | 842 |  |  |  | 0 | L |
| *nCR* | 13405 | 14117 | 713 |  |  |  | 0 |  |
| *tRNA^Ile^* | 14118 | 14183 | 66 |  |  |  | 6 | H |
| *tRNA^Gln^* | 14190 | 14258 | 69 |  |  |  | 7 | L |
| *tRNA^Met^* | 14266 | 14333 | 68 |  |  |  | 0 | H |
| *nad2* | 14334 | 15329 | 996 | 331 | ATG | TAA | 3 | H |
| *tRNA^Trp^* | 15333 | 15399 | 67 |  |  |  | -1 | H |
| *tRNA^Cys^* | 15399 | 15459 | 61 |  |  |  | 3 | L |
| *tRNA^Tyr^* | 15463 | 15528 | 66 |  |  |  | 0 | L |

^a^ TAa and Taa represent incomplete stop codons.

^b^ Numbers correspond to the nucleotides separating adjacent genes. Negative numbers indicate overlapping nucleotides.
