# Supplementary material for: Evolution and phylogeny of the mud shrimps (Crustacea: Decapoda) revealed from complete mitochondrial genomes
Source: BMC Genomics. 2012 Nov 16;13:631. doi: 10.1186/1471-2164-13-631 (PMC3533576; doi:10.1186/1471-2164-13-631)
Supplement: Additional file 5 — Location of genes in the mitochondrial genome of Neaxius glyptocercus. [file 1471-2164-13-631-S5.doc]

***Additional File 5*** *Location of genes in the mitochondrial genome of Neaxius glyptocercus*

| Gene | Position | | Size | | Codon | | Intergenic nucleotides^b^ | Strand |
| --- | --- | --- | --- | --- | --- | --- | --- | --- |
|  | From | To | Nucleotide | Amino acid | Start | Stop^a^ |  |  |
| *cox1* | 1 | 1536 | 1536 | 512 | ATG | TAA | 2 | H |
| *tRNA^Leu(CUN)^* | 1539 | 1601 | 63 |  |  |  | 2 | H |
| *tRNA^Leu(UUR)^* | 1604 | 1668 | 64 |  |  |  | 0 | H |
| *cox2* | 1669 | 2354 | 686 | 228 | ATG | TAa | 0 | H |
| *tRNA^Lys^* | 2355 | 2420 | 66 |  |  |  | 1 | H |
| *atp8* | 2422 | 2580 | 159 | 52 | ATG | TAA | -7 | H |
| *atp6* | 2574 | 3247 | 674 | 224 | ATG | TAa | 0 | H |
| *tRNA^Gly^* | 3248 | 3310 | 63 |  |  |  | -2 | H |
| *tRNA^Val^* | 3309 | 3376 | 68 |  |  |  | 0 | H |
| *nad3* | 3377 | 3730 | 354 | 116 | ATT | TAA | 4 | H |
| *cox3* | 3735 | 4526 | 792 | 263 | ATG | TAG | -2 | H |
| *tRNA^Ala^* | 4525 | 4590 | 66 |  |  |  | -1 | H |
| *tRNA^Arg^* | 4590 | 4652 | 63 |  |  |  | 0 | H |
| *tRNA^Asn^* | 4653 | 4718 | 66 |  |  |  | 0 | H |
| *tRNA^Ser(AGN)^* | 4719 | 4786 | 67 |  |  |  | 0 | H |
| *tRNA^Glu^* | 4787 | 4850 | 64 |  |  |  | -2 | H |
| *tRNA^Phe^* | 4849 | 4911 | 63 |  |  |  | -1 | L |
| *nad5* | 4911 | 6638 | 1728 | 575 | ATG | TAA | 0 | L |
| *tRNA^His^* | 6639 | 6702 | 64 |  |  |  | -1 | L |
| *nad4* | 6702 | 8042 | 1341 | 446 | ATG | TAA | -7 | L |
| *nad4L* | 8036 | 8338 | 303 | 100 | ATG | TAA | 2 | L |
| *tRNA^Thr^* | 8341 | 8405 | 65 |  |  |  | 0 | H |
| *tRNA^Pro^* | 8406 | 8470 | 65 |  |  |  | 3 | L |
| *nad6* | 8474 | 8980 | 507 | 168 | ATT | TAA | -1 | H |
| *cob* | 8980 | 10114 | 1135 | 378 | ATG | Taa | 0 | H |
| *tRNA^Ser(UCN)^* | 10115 | 10181 | 67 |  |  |  | 49 | H |
| *nad1* | 10231 | 11169 | 939 | 312 | ATA | TAA | 0 | L |
| *lrRNA* | 11170 | 12566 | 1397 |  |  |  | 0 | L |
| *srRNA* | 12567 | 13363 | 797 |  |  |  | 0 | L |
| *tRNA^Ile^* | 13364 | 13426 | 63 |  |  |  | 0 | L |
| *nCR* | 13427 | 13517 | 91 |  |  |  | 0 |  |
| *tRNA^Gln^* | 13518 | 13583 | 66 |  |  |  | -1 | L |
| *tRNA^Asp^* | 13583 | 13647 | 65 |  |  |  | -1 | H |
| *tRNA^Met^* | 13647 | 13713 | 67 |  |  |  | 0 | H |
| *nad2* | 13714 | 14706 | 993 | 330 | ATG | TAA | -2 | H |
| *tRNA^Trp^* | 14705 | 14770 | 66 |  |  |  | 1 | H |
| *tRNA^Cys^* | 14772 | 14837 | 66 |  |  |  | 2 | L |
| *tRNA^Tyr^* | 14840 | 14904 | 65 |  |  |  | 5 | L |

^a^ TAa and Taa represent incomplete stop codons.

^b^ Numbers correspond to the nucleotides separating adjacent genes. Negative numbers indicate overlapping nucleotides.
