# Supplementary material for: Evolution and phylogeny of the mud shrimps (Crustacea: Decapoda) revealed from complete mitochondrial genomes
Source: BMC Genomics. 2012 Nov 16;13:631. doi: 10.1186/1471-2164-13-631 (PMC3533576; doi:10.1186/1471-2164-13-631)
Supplement: Additional file 6 — Location of genes in the mitochondrial genome of Nihonotrypaea thermophilus. [file 1471-2164-13-631-S6.doc]

***Additional File 6*** *Location of genes in the mitochondrial genome of Nihonotrypaea thermophilus*

| Gene | Position | | Size | | Codon | | Intergenic nucleotides^b^ | Strand |
| --- | --- | --- | --- | --- | --- | --- | --- | --- |
|  | From | To | Nucleotide | Amino acid | Start | Stop^a^ |  |  |
| *cox1* | 1 | 1536 | 1536 | 511 | ATG | TAA | 2 | H |
| *tRNA^Leu(CUN)^* | 1539 | 1602 | 64 |  |  |  | 0 | H |
| *tRNA^Leu(UUR)^* | 1603 | 1666 | 64 |  |  |  | 1 | H |
| *cox2* | 1668 | 2357 | 690 | 229 | ATG | TAA | -1 | H |
| *tRNA^Lys^* | 2357 | 2421 | 65 |  |  |  | 1 | H |
| *atp8* | 2423 | 2581 | 159 | 52 | ATG | TAA | -7 | H |
| *atp6* | 2575 | 3249 | 675 | 224 | ATG | TAA | -1 | H |
| *tRNA^Gly^* | 3249 | 3314 | 66 |  |  |  | 0 | H |
| *tRNA^Val^* | 3315 | 3380 | 66 |  |  |  | 0 | H |
| *nad3* | 3381 | 3734 | 354 | 117 | ATT | TAA | 5 | H |
| *cox3* | 3740 | 4528 | 789 | 262 | ATG | TAG | -2 | H |
| *tRNA^Ala^* | 4527 | 4593 | 67 |  |  |  | -1 | H |
| *tRNA^Arg^* | 4593 | 4657 | 65 |  |  |  | -1 | H |
| *tRNA^Asn^* | 4657 | 4722 | 66 |  |  |  | 0 | H |
| *tRNA^Ser(UCN)^* | 4723 | 4789 | 67 |  |  |  | -1 | H |
| *tRNA^Glu^* | 4789 | 4857 | 69 |  |  |  | -2 | H |
| *tRNA^Phe^* | 4856 | 4922 | 67 |  |  |  | -1 | L |
| *nad5* | 4922 | 6619 | 1698 | 565 | ATG | TAA | 0 | L |
| *tRNA^His^* | 6620 | 6685 | 66 |  |  |  | -1 | L |
| *nad4* | 6685 | 8022 | 1338 | 445 | ATG | TAA | -7 | L |
| *nad4L* | 8016 | 8318 | 303 | 100 | ATG | TAA | 2 | L |
| *tRNA^Thr^* | 8321 | 8383 | 63 |  |  |  | 1 | H |
| *tRNA^Pro^* | 8385 | 8449 | 65 |  |  |  | 0 | L |
| *nad6* | 8450 | 8955 | 506 | 168 | ATT | TAa | -1 | H |
| *cob* | 8955 | 10089 | 1135 | 378 | ATG | Taa | 0 | H |
| *tRNA^Ser(UCN)^* | 10090 | 10154 | 65 |  |  |  | 19 | H |
| *nad1* | 10174 | 11112 | 939 | 312 | ATT | TAA | 0 | L |
| *lrRNA* | 11113 | 12469 | 1357 |  |  |  | 0 | L |
| *srRNA* | 12470 | 13251 | 782 |  |  |  | 0 | L |
| *tRNA^Ile^* | 13252 | 13317 | 66 |  |  |  | 0 | L |
| *nCR* | 13318 | 13863 | 546 |  |  |  | 0 |  |
| *tRNA^Gln^* | 13864 | 13930 | 67 |  |  |  | -2 | L |
| *tRNA^Asp^* | 13929 | 13994 | 66 |  |  |  | 2 | H |
| *tRNA^Met^* | 13997 | 14061 | 65 |  |  |  | 0 | H |
| *nad2* | 14062 | 15054 | 993 | 330 | GTG | TAA | -2 | H |
| *tRNA^Trp^* | 15053 | 15118 | 66 |  |  |  | -8 | H |
| *tRNA^Cys^* | 15111 | 15173 | 63 |  |  |  | 0 | L |
| *tRNA^Tyr^* | 15174 | 15238 | 65 |  |  |  | 2 | L |

^a^ TAa and Taa represent incomplete stop codons.

^b^ Numbers correspond to the nucleotides separating adjacent genes. Negative numbers indicate overlapping nucleotides.
