# Supplementary figures and images for: Evolution and phylogeny of the mud shrimps (Crustacea: Decapoda) revealed from complete mitochondrial genomes
Source: BMC Genomics. 2012 Nov 16;13:631. doi: 10.1186/1471-2164-13-631 (PMC3533576; doi:10.1186/1471-2164-13-631)

**Additional File 7**

**
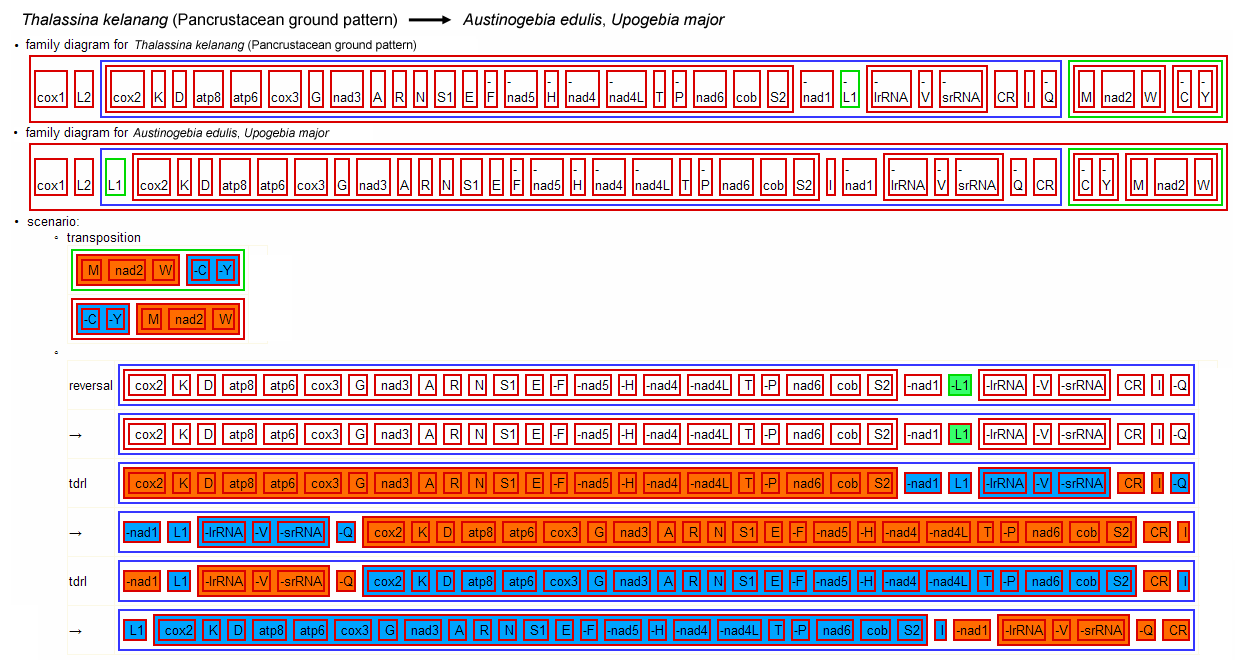
**

Supplement: Additional file 7 — Mitochondrial gene order rearrangement scenario of Austinogebia edulis, Upogebia major (Decapoda: Gebiidea) inferred by CREx. The elements in the blue shaded boxes are lost in the second copy, therefore the remaining copies are moved to the front. The elements in the red shaded boxes are lost in the first copy, therefore the remaining copies are moved to the back. [file 1471-2164-13-631-S7.doc]

**Additional File 8**

**
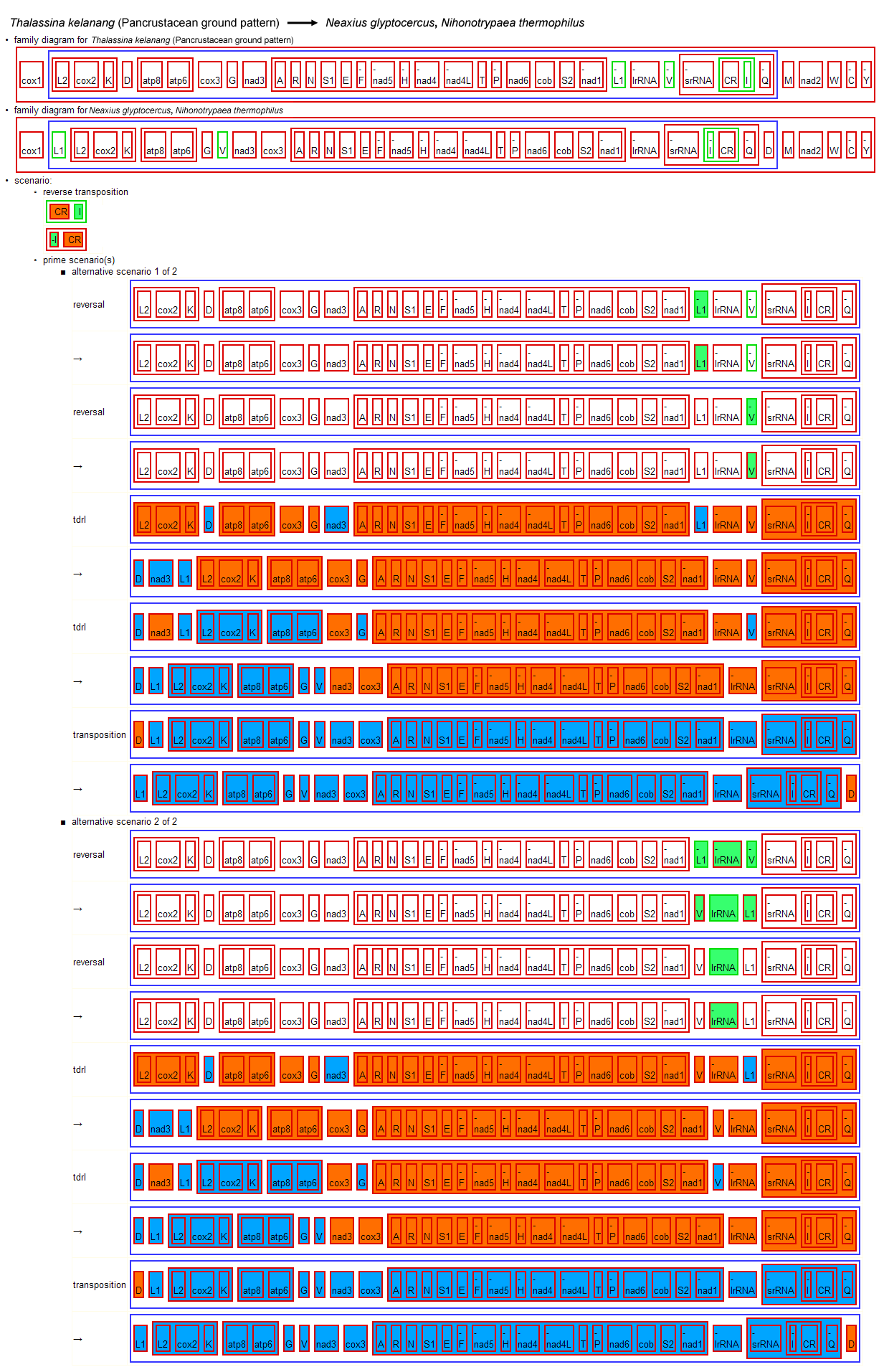
**

Supplement: Additional file 8 — Mitochondrial gene order rearrangement scenario of Neaxius glyptocercus and Nihonotrypaea thermophilus (Decapoda: Axiidea) inferred by CREx. The elements in the blue shaded boxes are lost in the second copy, therefore the remaining copies are moved to the front. The elements in the red shaded boxes are lost in the first copy, therefore the remaining copies are moved to the back. [file 1471-2164-13-631-S8.doc]
