# Supplementary material for: Evolution and phylogeny of the mud shrimps (Crustacea: Decapoda) revealed from complete mitochondrial genomes
Source: BMC Genomics. 2012 Nov 16;13:631. doi: 10.1186/1471-2164-13-631 (PMC3533576; doi:10.1186/1471-2164-13-631)
Supplement: Additional file 9 — Specific primers used in amplification of Austinogebia edulis and Upogebia major mitochondrial genomes. [file 1471-2164-13-631-S9.doc]

**Additional File 9** Specific primers used in amplification of *Austinogebia edulis* and *Upogebia major* mitochondrial genomes

| Species | Primer name | Sequence (5'-3') |
| --- | --- | --- |
| *Austinogebia edulis* | AEcox1F | GGA ATA ACA ATA GAC CGA ATA CC |
|  | AEcox1R | TAC GAG GAA AGG CTA TAT CAG GAG |
|  | AEnad5F | CAG GTA ATC AAG CCG AAA AAG G |
|  | AEnad5R | TAT TTA TGG CTG GAT TAG GTG C |
|  | AEcobF | GCC TTT TCA CCC ATA CTT CAC ATT |
|  | AEcobR | ATG TGA AGT ATG GGT GAA AAG GCA |
|  | AE16SF | TTC TTG TCC GAC CAT TCA TAC C |
|  | AE16SR | GCG TAA TTT TTC CTG AGA GCT C |
| *Upogebia major* | UMcox1F | TTG TTA CCG CCC ACG CCT TT |
|  | UMcox1R | AAA ATA CCT ATG TCA ACG GAA G |
|  | UMnad5F | GAT GAA TGA ACA AGA GAT GAA ACA GG |
|  | UMnad5R | AGC AGC ACC TAC TCC TGT TTC ATC |
|  | UMcobF | AAC GGG AGG AAA TAA CCC ACT G |
|  | UMcobR | AAT GTA AAG TAT GGG TGG AAG GGA AC |
|  | UM16SF | TTA TTT CTT GTC CGA CCA TTC A |
|  | UM16SR | AGA TTA AGG GGA CGA TAA GAC C |
