# Supplementary material for: Evolution and phylogeny of the mud shrimps (Crustacea: Decapoda) revealed from complete mitochondrial genomes
Source: BMC Genomics. 2012 Nov 16;13:631. doi: 10.1186/1471-2164-13-631 (PMC3533576; doi:10.1186/1471-2164-13-631)
Supplement: Additional file 10 — Specific primers used to amplification of the fragments which covered the gaps after sequencingThalassina kelanang, Neaxius glyptocercus and Nihonotrypaea thermophilus mitochondrial genomes. [file 1471-2164-13-631-S10.doc]

**Additional File 10** Specific primers used to amplification of the fragments which covered the gaps after sequencing *Thalassina kelanang*, *Neaxius glyptocercus* and *Nihonotrypaea thermophilus* mitochondrial genomes

| Species | Primer name | Sequence (5'-3') |
| --- | --- | --- |
| *Thalassina kelanang* | TK12s-R | TCA AGA AAG CGA CGG GCA ATA TGT ACA TA |
|  | TKbs-R | AGA GGT TGG TAA TAA CGG TGG CTC CTC A |
|  | TKc1s-R | CGG CTC ATG CTC CGA ATA CAA AGT ATA G |
|  | TKc2s-F | TAA TCG AAC AAT CCT GCC CAT AGA CAC CCA |
|  | TKd5s-R | GCT ATT TCT TTG GGT TTA GCT GGG TTG GCA |
| *Neaxius glyptocercus* | NGbs-R | AGA TAT GAA CTA ACG TAG CGG CAG CCA CGA |
|  | NGbs-F | AAT GAA TTT GAG GAG GAT TCG CAG TAG ATA |
|  | NGc1s-R | TAC CCA TAT CTA CAG AAG CTC CGG CAT GAG |
|  | NGc2s-F | AAC AGT CTT GCC CAT GAA TAC CCA GAT CCG |
|  | NGc3s-F | CTA CAC TCA GGC CGT TCA AAG ACT AGG GTT |
|  | NGc3s-R | CAG GCA GAT TAA CCT GCG CCT TAT GAA TGC |
|  | NGrRs-F | CCG AGA CAG TTG GTT CTT TGT CCG ACC GT |
|  | NGrRs-R | ACG GTC GGA CAA AGA ACC AAC TGT CTC GG |
| *Nihonotrypaea thermophilus* | NTbs-R | ATC TAC ATC CCT TCT CAC ACC CAG AGG A |
|  | NTc1s-R | TAC AGA AGC CCC AGC ATG TGC AAT TGA C |
|  | NTc2s-F | TAC TCA AAT TCG TGT TTT AGT GAG AGC AGC TG |
|  | NTrRs-F | CAG TTA AAT GTG TGC TTT CGT CGC CCC A |
